# Reduced Activity of HDAC3 and Increased Acetylation of Histones H3 in Peripheral Blood Mononuclear Cells of Patients with Rheumatoid Arthritis

**DOI:** 10.1155/2018/7313515

**Published:** 2018-10-03

**Authors:** Yan Li, Mi Zhou, Xiuying Lv, Lina Song, Di Zhang, Yan He, Mian Wang, Xue Zhao, Xiaoqing Yuan, Guixiu Shi, Dashan Wang

**Affiliations:** ^1^Department of Rheumatology, The First Affiliated Hospital of Xiamen University, Xiamen, Fujian 361003, China; ^2^The First People's Hospital of Yueyang, No. 39, Dongmaoling Road, Yueyang, Hunan 414000, China; ^3^Xiamen Xinkaiyuan Hospital, Xiamen, Fujian 361003, China; ^4^Fujian University of Traditional Chinese Medicine, Fuzhou 350122, China; ^5^Department of Medical Cosmetology, The First Affiliated Hospital of Xiamen University, Xiamen, Fujian 361003, China; ^6^Molecular Biology Research Center, Shandong Medical College, Linyi, Shandong 276000, China

## Abstract

Aberrant histone acetylation and deacetylation are increasingly thought to play important roles in the pathogenesis of rheumatoid arthritis (RA). However, limited data from studies about the activity of histone deacetylases (HDACs) and histone acetyltransferase (HAT) in RA are controversial. Those conflicting results may be caused by sample size, medication, and age- and sex-matched controls. The aim of this study is to investigate the expression and activity of class I HDACs (1–3.8) and their effects on histone acetylation in peripheral blood mononuclear cells (PBMCs) from RA patients. The expression of class I HDACs in PBMCs from RA patients was decreased in both mRNA and protein levels in comparison with HCs. The nuclear HAT activities were dramatically increased. Further, we found HDAC3 activity to be the most significantly reduced in overall reduction of HDACs in the RA group. The extent of total histone H3, but not H4, acetylation in PBMCs from RA patients was increased compared to that in healthy controls (HCs) (*p* < 0.01). In RA PBMCs, the activity and expression of class I HDACs are decreased, which is accompanied with enhanced HAT activity. An altered balance between HDAC and HAT activity was found in RA PBMCs.

## 1. Introduction

Rheumatoid arthritis (RA) is a chronic autoimmune disease mostly of unknown etiology, and a key characteristic of the disease is progressive destruction of articular cartilage [1]. Immune dysfunction, especially PBMCs secreting abnormal levels of pro- and anti-inflammatory cytokines infecting T helper (Th) cells differentiating towards Th1, Th2, Th17, and Treg cells, was involved in the evolution of the disease [2]. Accumulating evidence suggests that epigenetic changes, including histone modifications, play an important role in proinflammatory cytokine secretion in RA [3–5].

Acetylation and deacetylation of histones are controlled by histone acetyltransferases (HAT) and histone deacetylases (HDACs), respectively. HDACs promote a condensed chromatin state and restrain transcription factors and the RNA polymerase complex to gene promoter regions [6, 7]. There are at least eighteen HDACs in mammal cells that are categorized into four classes: class I (HDAC 1, 2, 3, and 8), class II (HDAC 4, 5, 6, 7, 9, and 10), class III (SIRT 1–7), and class IV (HDAC 11) [8]. Class I HDACs are ubiquitously expressed and enzymatically active to deacetylase histones and play an important role in modulating general cellular processes such as cell survival, apoptosis, proliferation, and differentiation [8, 9]. Therefore, in this study, we focus on class I HDACs.

Disruption of the physiological equilibrium between HAT, HDAC, and reader proteins can deregulate the transcription of key proinflammatory genes, such as tumor necrosis factor (TNF) and interleukin-8 (IL-8), leading to pathological conditions [10–12]. Since RA is an inflammatory disorder with overexpressed proinflammatory factors, therefore, an unbalance between HDAC and HAT activity may contribute to RA pathogenesis. Published data revealed relationships of HDACs and HAT with RA. However, results are controversial. Wada et al. reported that the level of histone H3 acetylation (H3ac) in the IL-6 promoter was significantly higher in RA synovial cells (SF) than in osteoarthritis (OA) SF [13]. Kawabata and colleagues reported that HDAC activity is increased in SF and in fibroblast-like synoviocytes (FLSs) of patients with RA and is accompanied by increased HDAC1 expression and synovial TNF-*α* production [14]. In stark contrast, another report showed reduced synovial HDAC activity in RASF compared with OASF, and the expression of HDAC1 and HDAC2 was correspondingly low [15]. Those results indicate that the exact role of HDACs in the pathogenesis of RA need to be further investigated.

In the few studies of overall HDAC activity in PBMCs from RA, discrepant results have also been reported [16, 17]. In RA, a global increase in HDAC activity was shown in PBMCs, and this activity was not affected after 12 weeks of etanercept therapy. By contrast, HAT activity levels did not differ between PBMCs of RA cases and HCs [17]. This report suggested that an unstable HAT/HDAC balance in RA may be due to saturation of HDAC activity. In contrast, Toussirot et al. reported that there are no major changes of both HDAC and HAT in PBMCs of RA patients [16]. Since the relationship of HDAC/HAT with RA is important in determining whether they can be therapeutic targets for RA, further studies to clarify are necessary.

Class I HDAC family members (HDAC1–3, 8) are thought to have a major role in regulating the expression of inflammatory genes and signaling pathways, both in arthritis models and in other inflammatory diseases [18–20]. Therefore, this study will focus on class I HDACs (1–3 and 8). Because RA is an autoimmune disease, and immune deregulation has been proven to be critical in its pathogenesis, we will study HDAC/HAT in PBMC of RA patients.

## 2. Materials and Methods

### 2.1. Patients and Healthy Donor Samples

Peripheral blood was obtained from 48 patients who were primarily diagnosed as RA and 48 HCs. All RA patients fulfilled the 1987 RA criteria of ACR and 2010 classification criteria of the ACR/EULAR [21]. None of the patients had ever taken disease-modifying antirheumatic drugs (DMARDs). Patients taking corticosteroids or vitamin D and those who had other diseases were excluded. The control group consisted of 48 age- and sex-matched HCs that did not have inflammatory or autoimmune disease. The study was performed in the Key Laboratory of Rheumatology and Immunology in Xiamen University after obtaining approval from the Ethics Committee of the First Affiliated Hospital of Xiamen University and informed consent from all of the patients.

Disease activity was scored in 28 joints (DAS28) [22], which have been widely used in clinical trials and for the assessment of patients in the clinic to monitor disease activity in patients with RA [23, 24]. The demographic and clinical features of HCs and patients with RA are summarized in Table 1.

### 2.2. Isolation of PBMC

PBMCs were isolated by Ficoll gradient centrifugation (Axis-Shield PoC AS, Oslo, Norway), washed, resuspended in phosphate-buffered saline (PBS), and treated with red blood cell lysis buffer to remove red blood cells. PBMCs were frozen in fetal bovine serum (Thermo Fisher) with 10% DMSO (Solarbio) and stored at −80°C.

### 2.3. Quantitative RT-PCR (qRT-PCR) Analyses

Total RNA was extracted from PBMCs using TRIzol Reagent (Ambion by Life Technologies) and was reverse-transcribed to cDNA with reverse transcription reagent kits according to the manufacturer's instructions (Bio-Rad, Hercules, CA, USA). The mRNA expression levels of class I HDACs and GAPDH were determined by real-time quantitative PCR. The primers used were HDAC1: forward 5′-AGACAGCTGTGGCCCTGGATAC-3′ and reverse 5′-CGGCAGCATTCTAAGGTTCTCAA-3′, HDAC2: forward 5′-TGCAGTTGCCCTTGATTGTGA-3′ and reverse 5′-ATCTGGACACCAGGTGCATGAG-3′, HDAC3: forward 5′-AGAGTGGCCGCTACTACTGTCTGAA-3′ and reverse 5′-TGGGTTGGTAGAAGTCCACTACCTG-3′, HDAC8: forward 5′-TGACGGAATGTGCAAAGTAGCAA-3′ and reverse 5′-TCAAATTTCCGTCGCAATCGTA-3′, and GAPDH: forward 5′-GTGAACCATGAGAAGTATGACAAC-3′ and reverse 5′-CATGAGTCCTTCCACGATACC-3′. 25 *μ*L SYBR Green II PCR reaction mixture containing 2 *μ*L of cDNA, 1 *μ*L of forward primer, 1 *μ*L of reverse primer, and 12.5 *μ*L of SYBR master mix (TaKaRa Shuzo) was used. Quantitative PCR was performed with the iQ™5 and MyiQ™ Real-Time PCR Detection Systems (Bio-Rad, Hercules, CA, USA). The mRNA expression was normalized to the expression levels of GAPDH, and relative expression was calculated with the 2^−ΔΔCt^ method.

### 2.4. Immunoblotting Analysis

Cells were lysed in RIPA buffer (Solarbio) including a complete protease inhibitor cocktail (Roche) to prevent proteolysis. After complete cell lysis, the cell debris was pelleted by centrifugation (12,000*g* (gravity) revolutions per minute at 4°C), and the supernatants containing total protein extracts were collected and stored at −80°C. These samples were subsequently used for Western blotting. An equal amount of protein (20 *μ*g) in each sample was separated on a 12.5% SDS-PAGE gel and was transferred to a nitrocellulose membrane. The membranes were incubated with primary antibodies overnight at 4°C. The following primary antibodies were used: HDAC1 (mouse monoclonal, 1 : 1000 dilution), HDAC2 (mouse monoclonal, 1 : 1000 dilution), HDAC3 (mouse monoclonal, 1 : 1000 dilution), HDAC8 (rabbit monoclonal, 1 : 1000 dilution), and GAPDH (rabbit monoclonal, 1 : 2000 dilution). Goat anti-mouse and goat anti-rabbit secondary IgG antibodies were used and incubated for 1 hour at room temperature. All antibodies were purchased from Cell Signaling Technology. Immunoreacted proteins were detected with a chemiluminescence reagent. Protein signals were quantified by ChemiDoc and after being normalized to GAPDH. Western blots were quantified using Image Lab.

### 2.5. HDACs and HAT Activity Assay

To separate the nuclear extracts from the cytoplasmic fraction of PBMCs, we used the nuclear/cytosol extraction kit (BioVision, USA). We next used the EpiQuik™ total HDAC activity and HDAC (1–3, 8) activity assay colorimetric kit (EpiGentek, Farmingdale, NY) and the HAT activity assay kit (Enzo Life Sciences, Koropi, Greece) to evaluate HAT and HDAC activities in PBMC nuclear extracts.

### 2.6. Total Histone Extraction and Measurement of Histone H3 and H4 Acetylation Levels

The EpiQuik Total Histone Extraction Kit was used to extract histone proteins for analysis. We used the colorimetric histone H3 and H4 acetylation assay (EpiQuik) to determine the extent of histone acetylation in PBMCs.

### 2.7. Detection of Apoptosis

PBMCs (2 × 10^6^/mL) were remained untreated or treated with TSA (300 nmol) for 24 h. The cells were washed in cold phosphate-buffered saline (1x PBS), the supernatant was discarded, and 10^6^ PBMCs were resuspended in 1 mL 1x annexin binding buffer. 5 *μ*L of annexin V and PI (BD Pharmingen™, San Diego, CA) were incubated in 100 *μ*L of resuspended PBMCs. After 15 min incubation at room temperature in the dark, 400 *μ*L of 1x annexin binding buffer was added and cells were acquired by a flow cytometer (Cytomics FC 500; Beckman Coulter, Fullerton, CA, USA). Results were analyzed and presented with CXP software (Beckman Coulter).

### 2.8. TNF and IL-6

Circulating TNF was evaluated in sera from patients using an ELISA assay (Quantikine assay, R&D Systems, Minneapolis, MN).

### 2.9. Statistical Analysis

Data were analyzed with Prism 5.01 software (GraphPad Software, San Diego, CA). The Mann–Whitney test was used to compare class I HDAC mRNA expression, HAT activity, HDAC activity, and total histone H3 and H4 acetylation levels between RA and HC. The correlation between the levels of class I HDACs and clinical characteristics was analyzed using the Spearman test. Results were expressed as the mean ± standard error of the mean (SEM). *p* values less than 0.05 were considered significant.

## 3. Results

### 3.1. Decreased mRNA and Protein Expression of Class I HDACs in PBMCs from Patients with RA

**Figure 1:**
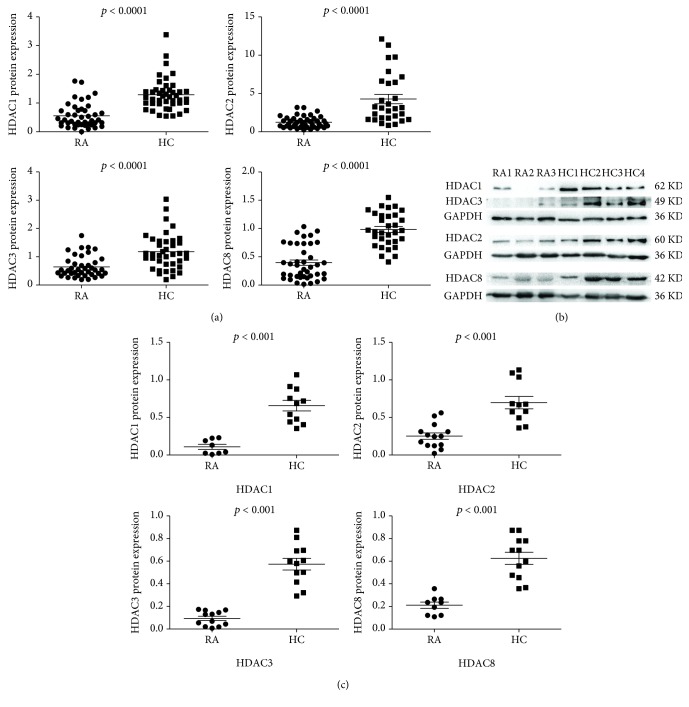
Decreased mRNA and protein expression of class I HDACs in PBMCs from patients with RA. (a) The mRNA expression of class I HDACs in the PBMCs of patients with RA (*n* = 46) and HCs (*n* = 46) was detected by real-time PCR. (b) Expression of class I HDAC proteins was detected using Western blot. (c) Relative protein expression of class I HDACs in PBMCs of patients with RA and HCs was normalized to GAPDH. The *p* values were determined using the Mann–Whitney test.

To assess the activity of HDACs in PBMCs from patients with RA, we measured the mRNA expression of HDACs in PBMCs from RA patients and HCs by real-time PCR. The data presented in Figure 1 shows that the mRNA expression of all class I HDACs (HDAC1, 2, 3, and 8) was significantly decreased in PBMCs of the RA group compared to those of the HC group (*p* < 0.0001) (Figure 1(a)). We performed Western blotting for total class I HDACs in PBMCs. The protein expression levels of total HDAC1, HDAC2, HDAC3, and HDAC8 were lower in RA PBMCs when compared with HC PBMCs (Figures 1(b) and 1(c)).

### 3.2. The Correlation between Class I HDAC Expression and RA Disease Characteristics

**Figure 2:**
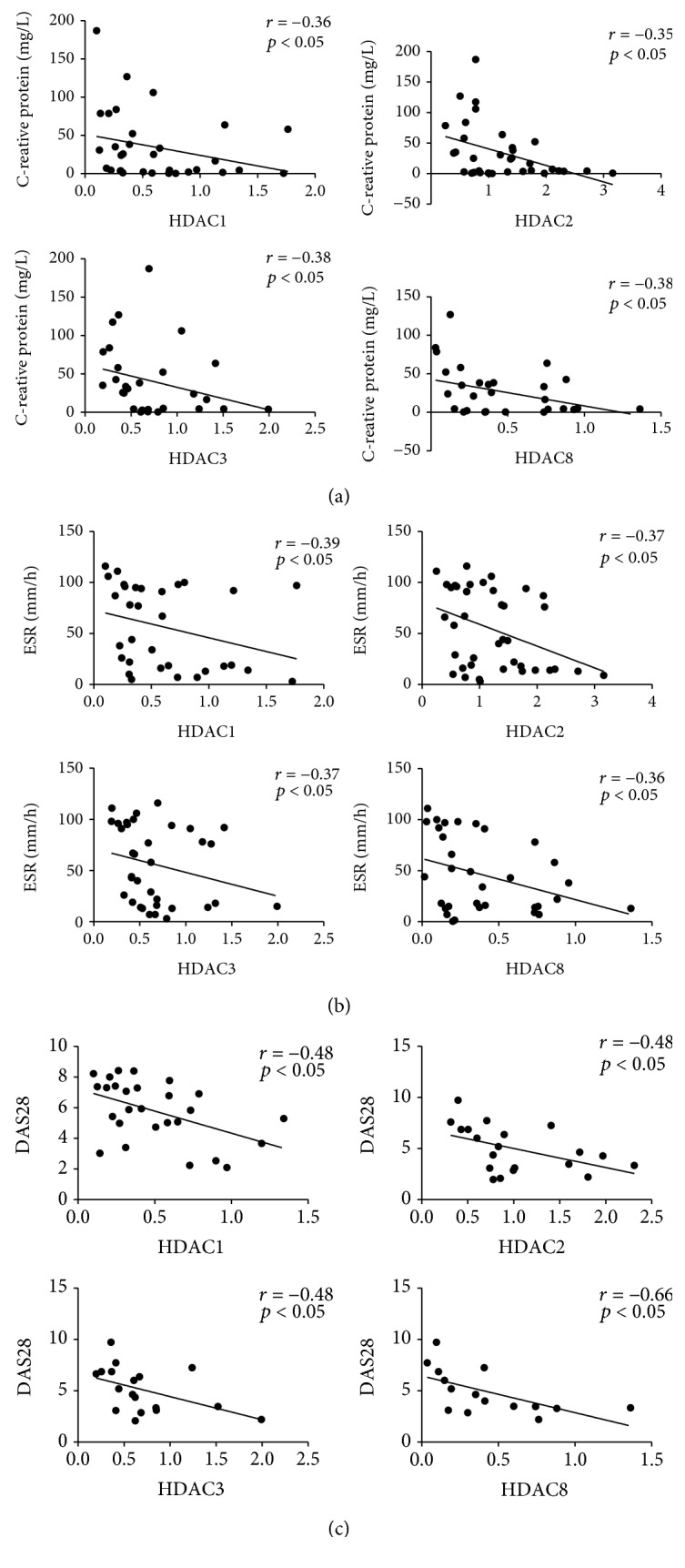
The mRNA expression of class I HDACs negatively correlates with CRP, ESR, and DAS28 levels. The correlations between the mRNA expression levels of class I HDACs and RA disease characteristics were determined by using the Spearman test and are shown for (a) C-reactive protein (CRP), (b) erythrocyte sedimentation rate (ESR), and (c) disease activity score (DAS28).

Our data show that class I HDAC expression was decreased in the PBMCs of patients with RA. To further study the association of class I HDACs with RA, we analyzed the relationship between the mRNA expression levels of class I HDACs and RA disease characteristics. We found strong negative correlations between the class I HDAC expression level and CRP, ESR, and DAS28 (Figure 2).

### 3.3. Increased Nuclear HAT Activity and Decreased Total Nuclear HDAC Activity in Patients with RA

**Figure 3:**
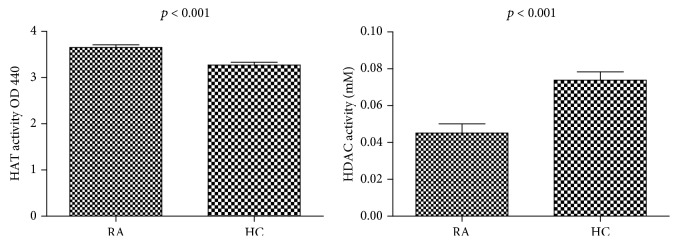
HAT and HDAC activities in the nuclear extracts of PBMCs in RA patients and HCs. HAT activity was significantly increased in RA patients compared with HCs. HDAC activity was significantly decreased in RA patients compared to that in HCs.

We further investigated HAT and HDAC activity in the nuclear extracts of PBMCs in patients with RA compared to HCs. HAT activity was significantly increased in RA patients (*n* = 26) compared to HCs (*n* = 24, *p* < 0.0001). Total nuclear HDAC activity in PBMCs in RA patients (*n* = 18) was significantly decreased compared with that of HCs (*n* = 18). (*p* ≤ 0.0001) (Figure 3). Therefore, there was an imbalance in the HDAC/HAT ratio that promoted histone hyperacetylation.

### 3.4. Nuclear HDAC3 Activity in the PBMCs of RA Patients Was Significantly Decreased Compared to That in HCs

**Figure 4:**
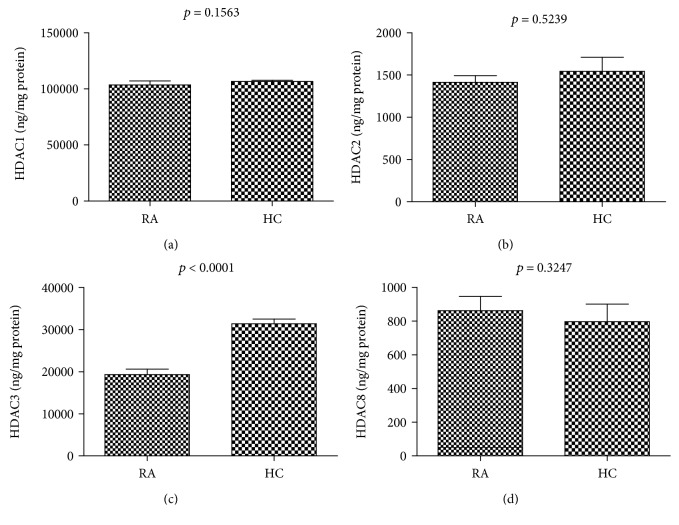
Individual class I HDACs activity in the nuclear extracts of PBMCs in RA patients and HCs. The HDAC3 activity level was significantly reduced in patients with RA compared to those of HC.

The level of HDAC3 activity in PBMCs from RA (*n* = 12) was significantly lower than that in PBMCs from the HCs (*n* = 13, *p* < 0.0001) (Figure 4). There was no significant difference in HDAC1, HDAC2, or HDAC8. We therefore propose that the total HDAC activity was reduced mainly due to a decrease in HDAC3.

### 3.5. Increased Total Histone H3 Acetylation in the PBMCs of Patients with RA Compared to HCs

**Figure 5:**
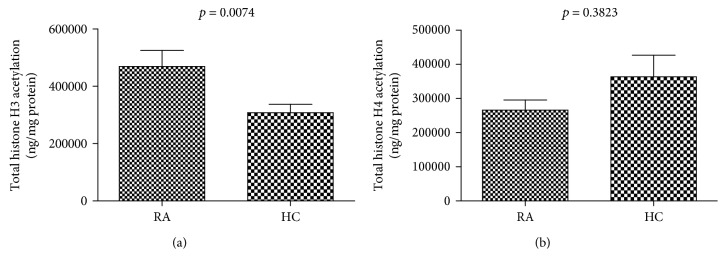
The level of total histone H3 and H4 acetylation in PBMCs of RA patients compared to HCs. The total histone H3 and H4 acetylation levels were measured using the colorimetric histone H3 and H4 acetylation assay. (a) A significant increase in total histone H3 acetylation levels in RA (*n* = 12) PBMCs compared to HCs (*n* = 10). (b) Lower but not statistical significance in the total histone H4 acetylation levels between RA patients and HCs.

The nucleosome includes H2A, H2B, H3, and H4. Histones undergo posttranslational modifications, which alter their interaction with DNA and histones. The H3 and H4 histones have long tails that protrude from the nucleosome, which can be covalently modified through methylation, acetylation, and phosphorylation more easily than H2A and H2B [25]. Acetylation of H3 and H4 histone proteins was measured in PBMCs from patients with RA compared to those from HCs (Figure 5). The data showed an increase in total histone H3 acetylation in the PBMCs of patients with RA. However, there was no significant difference in total H4 histone acetylation levels between HCs and RA.

### 3.6. Detection of Apoptosis in PBMCs from HC and RA

**Figure 6:**
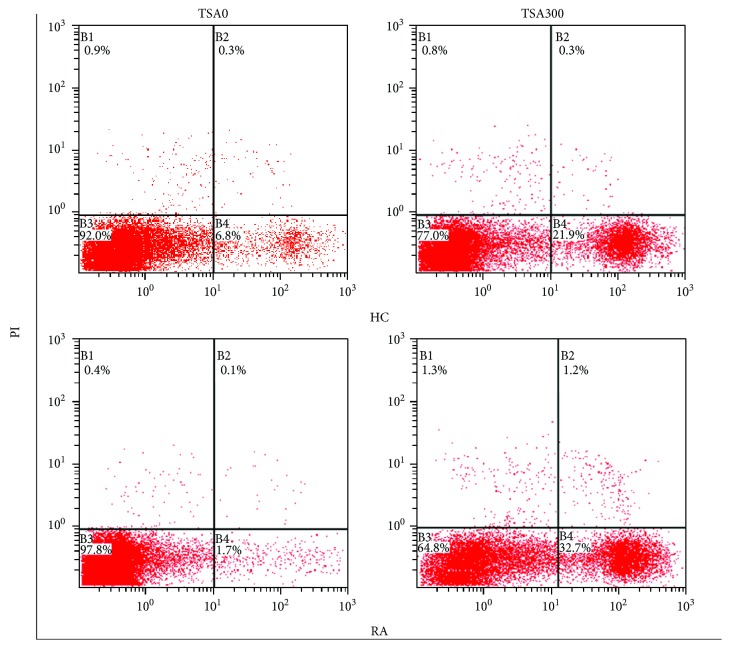
Apoptosis of PBMC cells from HC and RA treated with 0 or 300 nmol/L of TSA for 24 hours.

Apoptosis was then assessed in HC and RA PBMCs treated with TSA using annexin-V and PI assay. TSA triggered apoptosis in PBMCs (Figure 6).

### 3.7. The Correlation about TNF-*α* Expression and HDAC Activity in RA Patients

**Figure 7:**
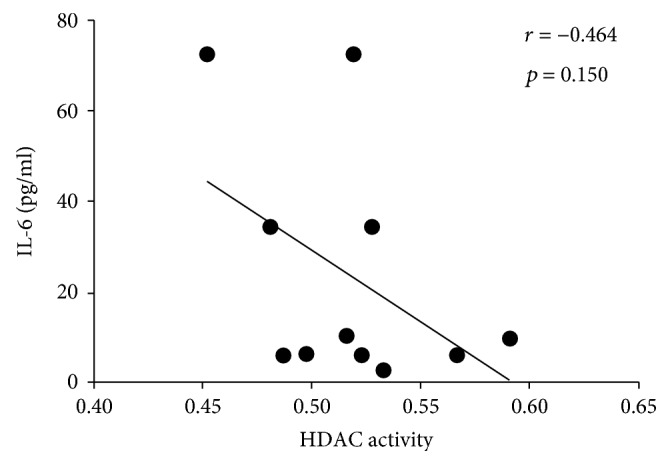
The correlation about IL-6 expression and HDAC activity in RA patients.

**Figure 8:**
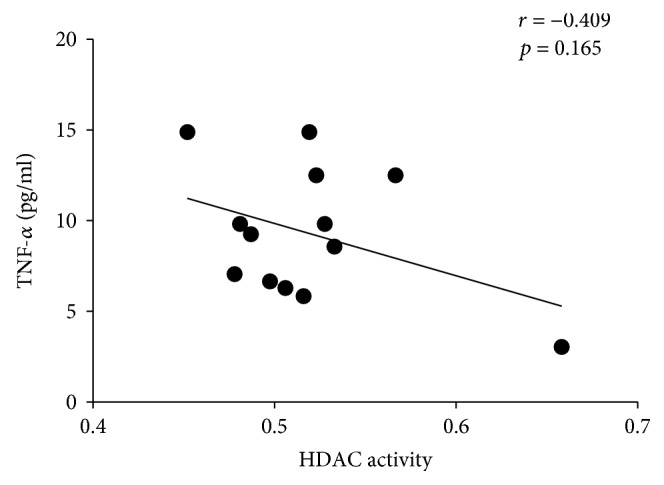
The correlation about TNF-*α* expression and HDAC activity in RA patients.

We also detected the IL-6 and TNF-*α* expression in RA serum by ELISA. HDAC activity has a negative correlation with IL-6 (*n* = 11) and TNF-*α* (*n* = 13), although not statistically (Figures 7 and 8). HDAC activity decrease in RA as a factor of hyperacetylation can contribute to the activation of gene coding for proinflammatory cytokines and thus to the pathogenesis of RA.

## 4. Discussion

Specific class I HDAC family members have a major role in regulating the expression of inflammatory genes and signaling pathways, both in arthritis models and in other inflammatory diseases [18–20]. Here, we determined the expression of class I HDACs in the PBMCs of patients with RA and investigated the correlation between HDAC expression and disease characteristics (ESR, CRP, and DAS28). Our results indicate that the mRNA and protein expressions of all class I HDACs in PBMCs were significantly decreased in patients with RA compared to HCs. In addition, we observed a negative correlation between the mRNA expression of class I HDACs and disease characteristics. Taken together, this suggests that class I HDACs might play an important role in RA pathogenesis.

Few studies have evaluated the equilibrium between total HAT and HDAC activity in RA [26–28]. Our results showed that the expression of class I HDACs in RA patients was reduced; thus, it is speculated that the activity of class I HDACs in RA patients would be decreased, and they will further affect the balance of HDACs to HAT activity. We found that the total activity of HDACs was significantly decreased; as expected, the activity of HAT was increased compared to HCs. This fits with our hypothesis that the HDAC-to-HAT ratio is disturbed in RA.

Although there have been several studies of HDAC activity in SF and FLs [14, 15], limited data were available on the levels of HAT or HDAC activities in PBMC extracts from patients with RA. Gillespie et al. evaluated HAT and HDAC activities in the PBMCs of a small number of patients with RA (*N* = 8) and found increased HDAC activity but no changes in the levels of HAT compared to HC [17]. Toussirot et al. found that HAT and HDAC activities were altered in ankylosing spondylitis while there were no major changes in RA [16]. The controversial conclusions drawn from these studies may be due to small sample size, disease stages, age- and sex-matched controls, and medications. In this study, we measured the activity of HDAC and HAT in PBMCs from a relatively larger group of patients (*n* = 48) with RA, and our results yielded divergent conclusions.

Several studies reported HDAC activity in synovial tissue samples, one study showed decreased HDAC activity [15], and another suggested that HDAC activity was increased in patients with RA compared to those with OA [14, 29]. Despite that these two studies had small sample sizes, one study used age unmatched controls (The normal controls comprised of a small sample size of men aged approximately 19 years, while the patient group comprised mostly of female RA with an average age of 65 years) [14]. RA is considered as a systemic autoimmune disease, and synovial tissue change is a local pathology of the systemic disease; therefore, the HDAC activity changes in RASF may not represent those in PBMCs. As a systemic autoimmune disease, the pathogenesis of RA is mainly immune deregulation. In this point of view, immune cells, such as T, B, and monocytes, are major players in the pathogenesis of RA [30]. The traditional treatments for RA are immune suppression reagents, such as DMARDs. Since the majority of PBMCs are lymphocytes and monocytes, studying the role of HDAC/HAT in PBMCs of RA might be more relevant to the pathogenesis of RA. In this study, we recruited an age- and gender-matched control group, with a relatively larger sample size of RA which are all DMARD-free, and the HDAC activity and levels of HAT in PBMC were studied.

Currently, there is no available data on the activity of each individual member of class I HDACs in RA. We found that the activity of HDAC3 was significantly decreased compared to HDAC1, 2, and 8 (Figure 4). Thus, we speculate that the overall reduction in HDAC activity was mainly due to a reduction in HDAC3 activity. Our data suggest that either the expression or the function of HDAC3 may be altered in RA PBMCs, or the key pathways associated with HDAC3 activity are preferentially utilized in RA PBMCs. Since the Th17/Treg axis is critical in the pathogenesis, consistent with our results, studies with HDAC3 knockout mice demonstrated that HDAC3 plays important roles in development and function of Tregs and the differentiation of Th17 [31, 32]. Periphery CD4 T cells of HDAC3 knockout mice were skewed toward RORgammat(+) IL-17-producing Th17 cells, leading to inflammatory bowel disease [31]. Another study demonstrated that FOXP3^+^ regulatory T cell development and function require histone/protein deacetylase 3, and HDAC3-deficient mice died from autoimmunity by 4–6 weeks of age [32].

Low HDAC activity in RA has previously been shown to result in hyperacetylation, which can contribute to the activation of genes coding for proinflammatory cytokines, including TNF-*α* and interleukin-8 (IL-8), and thus to the pathogenesis of RA [5, 15, 29, 33]. Our data demonstrated that the activity of HDAC especially HDAC3 is significantly reduced in RA PBMCs, which results in hyperacetylation of H3 histone, supporting the role of HAT as potential targets in RA. Curcumin, a HAT inhibitor, significantly suppressed inflammatory signaling by reducing the level of H3ac in the IL-6 promoter, as well as IL-6 mRNA expression and IL-6 protein secretion by RASFs [13]. This suggests that chromatin structure is in an open or loose state in the IL-6 promoter in RASFs. Delphinidin (DP), which specifically inhibited the HAT activities of p300/CBP, may be useful in preventing inflammatory arthritis by blocking anti-inflammatory signaling: p65 acetylation and this compound [34]. Taken together, it is suggested that hyperacetylation of histone H3 induces the increase in inflammation factors and thereby participates in the pathogenesis of RA.

## 5. Conclusion

In conclusion, our results suggest that the decreased expression and activity of class I HDACs disturbed the balance of HDACs and HAT activity in patients with RA. This hyperacetylation status may support proinflammatory processes and ultimately contribute to RA pathogenesis. The HDAC activity levels and histone H3 acetylation status in PBMCs have potential as a biomarker of disease activity. It also indicates that HAT inhibitions other than HDAC inhibitors might be a potential target in RA therapy.

## Figures and Tables

**Table 1:** Demographic and clinical characteristics of patients with RA and HCs.

	RA patients (*n* = 48)	HCs (*n* = 48)
Age, mean (range) years	44.1 (24–69)	42.9 (26–63)
No. of women/no. of men	38/10	36/12
Anticyclic citrullinated protein antibodies (RU/mL)	57 (3–200)	
Rheumatoid factor (IU/mL)	287 (11–1580)	
C-reactive protein (mg/L)	52.3 (3.6–127.4)	0.8 (0.2–1.9)
DAS28 score	4.92 (1.97–8.73)	
Disease duration, mean (range) months	10.5 (1.3–17.8)	

DAS28 = 28-joint disease activity score.

## Data Availability

The data used to support the findings of this study are available from the corresponding author upon request.
